# Construction Notes

**Published:** 1907-07-27

**Authors:** 


### CONSTRUCTION NOTES.

The new workhouse infirmary at Newport, Salop, now in course of erection, provides for 29 beds in four wards. The two large wards, male and female, are for ordinary cases; the third ward is for maternity cases; while the fourth serves as an isolation room. The cost is £4,015.

The committee of the North Riding Infirmary reports that it has been found possible to undertake the special repairs and renovations. The alterations to the accident and medical wards, the provision of the new female medical ward, and the erection of the administrative buildings at the front of the infirmary, as a memorial to the late Sir Bernard Samuelson, are now completed. These buildings constitute a most valuable gift to the infirmary, and have enlarged its capacity by nine beds.

The Madras Government have sanctioned estimates aggregating to Rs. 60,836 for improvements to the maternity Hospital, Madras. The improvements include the construction of an operating-theatre, linen-rooms, and quarters for the assistant matrons and a labour ward for infectious cases. An estimate amounting to Rs. 29,100 has also been sanctioned for constructing a hostel with outhouses for the Government College at Rajahmundry, Godavery district.

The new wing of the Glasgow Samaritan Hospital, containing 50 additional beds, with fully equipped operating-rooms on each floor, has now been completed and occupied, and the old wards are undergoing renovation. In order to enable the enlarged hospital to be properly worked, it has been found necessary to provide a new entrance hall and also additional accomodation in the administrative block. The directors hope that the entire building, with the new wing, will be ready for opening on an early date. The enlarged hospital will have accommodation for 86 beds, and will be one of the largest women's hospitals in the United Kingdom. The cost of building and furnishing the new wing and operating-rooms, together with the other additions and alterations, amounts to over £12,000, making the total cost of the whole hospital buildings, including the nurses' home and dispensary, with equipment and furnishings, about £40,000. The extensions have rendered necessary additional buildings and plant, estimated to cost not less than £5,000.

Important extensions and alterations have been carried out in connection with the Ingham Infirmary, South Shields, during the past year. A nurses' home has been erected, a new operating theatre has been constructed, and a new dispensary, complete and up to date in every detail, has just been added.

The extensions and improvements in connection with the North Devon Infirmary have recently been opened. The casualty department on the basement has been remodelled, and by means of a trolley patients can be conveyed direct to a lift, and thence to the operating theatre or to any ward in the building. The men's and children's side of the institution is divided into three wards, in each of which two wards have now been thrown into one. On the women's side bathroom accommodation has been provided. An apartment adjoining the principal women's ward in the main building has been adapted as a ward for four beds, for use in cases of emergency. On the second floor sanitary accommodation has been provided for the nurses and servants, in a new exterior annexe, directly over that of the women's ward. The sanitary towers have been built of stone, with solid concrete floors carried on steel joints. The walls are lined with white tiles, and the partitions are of Tiltman's patent bricks, glazed on both sides. A feature is that the whole of the corners are rounded, even the slightest accumulation of dust being thereby rendered impossible. At the rear of the infirmary a new ward has been built to contain two beds (with its own sanitary accommodation) and a nurses' duty-room, by which the authorities will be able to isolate any cases of infectious disease which may arise in the hospital. The ward hitherto used as the children's ward has now been converted into nurses' rooms. The operating theatre has been entirely remodelled. A portion of the operating theatre has been partitioned off as a sterilising-room. On the basement the casualty, waiting-rooms, and surgery have been rearranged, whilst a new dark room has been fitted up for eye cases. The new patients' lift has been fitted with a safety apparatus, so that in the remote event of the rope breaking no danger could possibly arise. The heating apparatus has been thoroughly overhauled, special chambers being provided for drying the mattresses and for heating the bath and lavatory water. The contractor for the building was Mr. William Slee, and the capable architect is Mr. J. C. Southcombe.

